# Global Ocean Sampling Collection

**DOI:** 10.1371/journal.pbio.0050083

**Published:** 2007-03-13

**Authors:** Hemai Parthasarathy, Emma Hill, Catriona MacCallum

## Abstract

This issue of *PLoS Biology* features research articles from the J. Craig Venter Institute's Global Ocean Sampling expedition, as well as accompanying material highlighting the promise, significance, and challenges of metagenomics.

**Figure:**
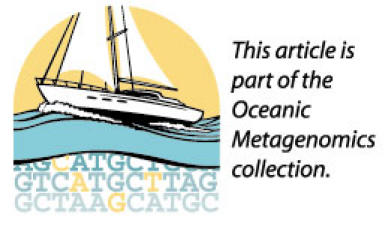


Today, *PLoS Biology* publishes landmark metagenomics papers from the J. Craig Venter Institute's Global Ocean Sampling expedition [1–3]. These papers describe the initial analyses of several gigabasepairs' worth of sequence data from oceanic microbes collected during the *Sorcerer II* expedition, as the ship made her way down from Canada, through the Panama Canal, and finally out beyond the Galapagos Islands well into the tropical Pacific and the South Pacific Gyre. Results from the first foray of this research mission into the Sargasso Sea were published three years ago [4]. As described in the accompanying Synopsis [5], the new voyage has added information from multiple biomes and several-fold more data.

Analysis of these data poses not only scientific challenges [6], but also significant legal hurdles. Craig Venter is no stranger to issues of intellectual property—his previous incarnation as the president of Celera saw him embroiled in controversy over the decision to “privatize” aspects of his company's work in sequencing the human genome. Now, at the head of the Global Ocean Sampling project, Venter finds himself on the side of greater accessibility, negotiating the claims of individual governments on the genomic wealth within their waters. In particular, as of this writing, there is an active negotiation with the Ecuadorian government (which has seen more than one change of power since the expedition began) over restricting commercial reuse of these data. Henry Nicholls describes this tangled legal landscape in an accompanying Feature [7].

Although extensive in scope, the papers presented here only touch the surface of the wealth of information to be gleaned from these data, which are freely available for all to explore from their desktops: the trace reads and processed data have been deposited in the National Center for Biotechnology Information's Trace Archive (http://www.ncbi.nlm.nih.gov/Traces) (with the exception of that fraction of the trace data acquired from Ecuadorian coastal waters), annotated with extensive geographical and physicochemical metadata. The assemblies and associated annotated peptides will be delivered to GenBank (http://www.ncbi.nlm.nih.gov/Genbank) around the time of publication, and will become available after GenBank has processed them. More immediately, and potentially more usefully, these data are also freely available through a specially built database, CAMERA—Cyberinfrastructure for Advanced Marine Microbial Ecology Research and Analysis (http://camera.calit2.net)—which provides greater annotation and analysis capabilities [8]. (CAMERA was funded by the Gordon and Betty Moore Foundation, which also supports PLoS.)

The proponents of open-access publishing, ourselves included, often cite as an inspiration the power that open access to DNA sequence databases has had in transforming scientific discovery. As our founders noted in the inaugural issue of *PLoS Biology*, “With great foresight, it was decided in the early 1980s that published DNA sequences should be deposited in a central repository, in a common format, where they could be freely accessed and used by anyone. Simply giving scientists free and unrestricted access to the raw sequences led them to develop the powerful methods, tools, and resources that have made the whole much greater than the sum of the individual sequences…. Now imagine the possibilities if the same creative explosion that was fueled by open access to DNA sequences were to occur for the much larger body of published scientific results.” [9]

But the publishing reality in genomics research has been less inspiring. Although sequence data are publicly available and free to be reused by the community, the same creative license has not yet been awarded to the key papers resulting from the major genome projects, which are commonly published in subscription-based journals. Many of these genomics papers are “freely” available from publisher Web sites, but their use remains restricted, and to claim that freedom to read an article is the main benefit of open access is to miss the promise inspired by DNA sequence databases.

While we and other open-access journals have both enjoyed and been grateful for strong support from the genomics community, we are also disappointed that authors of landmark genomics papers, who adamantly support open access to sequence data, have not taken the opportunity to provide further leadership for their community by promoting open access to the scientific literature. We encourage all researchers to apply the same standards to their papers as they would to their data, regardless of the publisher. As Jensen et al. stated in a recent review about the benefits of text mining for the scientific community, “It is the restricted access to the full text of papers…that is currently the greatest limitation…” [10].
